# ^18^F-SynVesT-1 PET/MR Imaging of the Effect of Gut Microbiota on Synaptic Density and Neurite Microstructure: A Preclinical Pilot Study

**DOI:** 10.3389/fradi.2022.895088

**Published:** 2022-05-23

**Authors:** Sue Y. Yi, Ali Pirasteh, James Wang, Tyler Bradshaw, Justin J. Jeffery, Brian R. Barnett, Nicholas A. Stowe, Alan B. McMillan, Eugenio I. Vivas, Federico E. Rey, John-Paul J. Yu

**Affiliations:** ^1^Neuroscience Training Program, Wisconsin Institutes for Medical Research, University of Wisconsin–Madison, Madison, WI, United States; ^2^Department of Radiology, University of Wisconsin School of Medicine and Public Health, Madison, WI, United States; ^3^Department of Medical Physics, University of Wisconsin School of Medicine and Public Health, Madison, WI, United States; ^4^Department of Bacteriology, University of Wisconsin–Madison, Madison, WI, United States; ^5^Gnotobiotic Animal Core Facility, Biomedical Research Model Services, University of Wisconsin–Madison, Madison, WI, United States; ^6^Department of Biomedical Engineering, University of Wisconsin–Madison, Madison, WI, United States; ^7^Department of Psychiatry, University of Wisconsin School of Medicine and Public Health, Madison, WI, United States

**Keywords:** gut microbiome, synaptic density, brain microstructure, SynVesT-1, NODDI, PET/MRI

## Abstract

The gut microbiome profoundly influences brain structure and function. The gut microbiome is hypothesized to play a key role in the etiopathogenesis of neuropsychiatric and neurodegenerative illness; however, the contribution of an intact gut microbiome to quantitative neuroimaging parameters of brain microstructure and function remains unknown. Herein, we report the broad and significant influence of a functional gut microbiome on commonly employed neuroimaging measures of diffusion tensor imaging (DTI), neurite orientation dispersion and density (NODDI) imaging, and SV2A ^18^F-SynVesT-1 synaptic density PET imaging when compared to germ-free animals. In this pilot study, we demonstrate that mice, in the presence of a functional gut microbiome, possess higher neurite density and orientation dispersion and decreased synaptic density when compared to age- and sex-matched germ-free mice. Our results reveal the region-specific structural influences and synaptic changes in the brain arising from the presence of intestinal microbiota. Further, our study highlights important considerations for the development of quantitative neuroimaging biomarkers for precision imaging in neurologic and psychiatric illness.

## Introduction

Interactions between commensal gut bacteria and the central nervous system (CNS) profoundly impact brain structure and function (1). Clinical and epidemiological studies have shown changes in gut microbiota are associated with neurologic, neurodegenerative, and psychiatric disorders (2–6) and collectively reflect the relationship between intestinal microbiota and neurologic and psychiatric health throughout the lifespan. Although the causal links between altered gut microbiota and neurologic and psychiatric illness remains unknown, cellular and molecular studies of germ-free (GF) and gnotobiotic animals have shown disruptions to neurogenesis (7), cortical myelination (8) and microglia-mediated synaptic pruning (9) as well as deficits in microglia maturation (9) and fear extinction learning (10). However, despite a growing understanding of the microbiota-gut-brain axis and their clinical correlates, our ability to characterize, quantify, and track microbiota-gut-brain interactions in clinical practice remains limited.

These limitations largely stem from the absence of clinically accessible measures of brain structure and function that are both neurobiologically salient and sensitive to the composition of gut microbiota. Many currently employed strategies use structural techniques (qT1, T2) to study the relationship between gut microbial composition and the brain, but these methods do not provide meaningful insights into neurobiology. Additionally, even though previous work has demonstrated strong associations between altered gut microbiomes and psychiatric illness, the small number of imaging studies exploring the link between the gut and the brain are confounded by an inability to distinguish neuropathological changes occurring as a result of the disease process from those potentially arising from the gut microbiota themselves (11). And critically, although studies of germ-free (GF) and gnotobiotic animals have uncovered the molecular and cellular effects of commensal gut bacteria on the brain, little is known about the contribution of gut microbiota to baseline measures of neural microstructure or brain function, which further hampers our collective ability to understand and ultimately assess the impact of altered gut microbiota in the disease state.

Guided by previous evidence demonstrating that GF and conventionally colonized mice possess significant differences in neurite morphology (12) and that alterations in synaptic density, a crucial marker of overall brain function, is associated with neurodevelopmental disorders such as autism spectrum disorder (ASD) and psychiatric illnesses such as schizophrenia and major depressive disorder (13–15), we performed ^18^F-SynVesT-1/NODDI (neurite orientation dispersion and density imaging) PET/MRI on GF and gnotobiotic mice to acquire both structural (neurite density and morphology) and synaptic measures of the brain that are both quantitative, complementary, and translationally relevant. Further, as a corollary, we sought to determine the degree to which resident gut microbes contribute to brain microstructure and synaptic density. Lastly, as both ^18^F-SynVesT-1 PET and NODDI MRI are techniques that can be performed on clinical timescales, our findings additionally highlight the clinical translational potential of ^18^F-SynVesT-1/NODDI PET/MRI for imaging the structural and synaptic correlates of the gut microbiome in human health and disease.

## Materials and Methods

### Animals

All experimental work was performed in accordance with animal protocols approved by the institutional animal care and use committee at our institution (Protocol Nos. M005899, M05599, and M05532). All GF C57BL/6 mice were maintained in a controlled environment in plastic flexible film gnotobiotic isolators under a strict 12 h light/dark cycle and received sterilized water and standard chow (LabDiet 5021; LabDiet, St Louis, MO) *ad libitum*. Sterility of GF animals was confirmed by incubating freshly collected fecal samples under aerobic and anaerobic conditions using standard microbiology methods and PCR analysis of the bacterial 16S DNA region. Humanized gnotobiotic (HG) mice were generated by oral gavage of GF mice with well-characterized human gut bacterial isolates (16) at 8 weeks of age, which contained a representative core community of eight species that are commonly found in the human microbiota (*Anaerotruncus colihominis, Bacteroides caccae, Bacteroides thetaiotaomicron, Clostridium symbiosum, Collinsella aerofaciens, Coprococcus comes, Providencia stuartii* and *Ruminococcus torques*). All HG animals are bred and maintained under specific pathogen free conditions and maintained on an irradiated standard chow diet (TD.2918; Envigo, Madison, WI). The successful transplantation of this community was confirmed using community profiling by sequencing (COPRO-Seq) (16). All animals were born and raised at our institution. The age of experimental animals were P55 germ-free (*n* = 5); gnotobiotic (*n* = 5); and P90 germ-free (*n* = 4) and gnotobiotic (*n* = 4). Only male animals were used to control for estrous effects. Imaging sample size estimations are based on a Markov Chain Monte Carlo approach (17) and informed by our previously published work (18–21) with power analyses indicating observed effect size values of d = 2.5 or greater given low standard deviation between within-group replicates (σ between 0.01 and 0.001), thus validating sample sizes of 4 or greater replicates per group.

### ^18^F-SynVesT-1 Generation

^18^F-SynVesT-1 was generated as previously described (22). Briefly, cyclotron produced ^18^F-fluoride ions were first trapped onto a quaternary ammonium anion (QMA) exchange cartridge, eluted, dried, and mixed with a fresh prepared solution of 2.5 mg SynVesT-1 precursor, 5 mg copper triflate, and 8 μL of pyridine in 0.5 mL of dimethylacetamide; the reaction vial was then heated at 110°C for 20 min, cooled to 50°C and purified with a Luna C18 (2) 10 × 250 mm (5 μm) column. The purified product was collected into a dilution vial containing 40 mL of water and with a C18 cartridge, subsequently trapped, washed, and eluted with 1 mL of ethanol injection into a product vial containing 5 mL of 0.9% sodium chloride. The product purity was >99% and decay uncorrected yield was 16%.

### PET/CT Imaging and PET ROI Analysis

At post-natal days 55 and 90 (P55 and P90), age- and sex-matched male GF mice and HG mice were imaged under isoflurane gas anesthesia on a small animal PET/CT scanner (Siemens Inveon Hybrid). CT was acquired for attenuation correction and anatomic localization. Dynamic PET imaging was carried out under anesthesia for 45 min. 5.5 MBq ± 0.4 (range 4.7–6.0) of ^18^F-SynVesT-1 was administered intravenously through the lateral tail vein. The 45-min ^18^F-SynVesT-1 PET acquisition was reconstructed into 1-min-per-frame images, starting at *t* = 60 s using 3-dimensional ordered-subset expectation maximization (OSEM, 2 iterations, 16 subsets) followed by a maximum a posteriori probability (MAP) algorithm. The PET and CT images were reconstructed at 128 × 128 × 159 (0.78 × 0.78 × 0.8 mm) and 480 × 480 × 635 (0.21 × 0.21 × 0.21 mm), respectively. PET images were converted to standardized uptake values (SUV). 3D regions of interest (ROIs) were manually placed over the whole brain and SUV_mean_ (herein referred to as SUV) at each frame was recorded using MIM (MIM Software Inc., Cleveland OH). One GF mouse was excluded from the ^18^F-SynVesT-1 analysis due to substantial motion. SUVs at each time point were averaged within each mouse cohort and ^18^F-SynVesT-1 time-activity curves were generated for each cohort. The area under the curve (AUC) for GF and HG cohorts were calculated at the peak stabilized interval (4.5–10.5 min).

After 24 h, the same mice were administered 10.7 MBq ± 0.2 (range 10.4–11.1) of ^18^F-flurodeoxyglucose (FDG) through the lateral tail vein. Sixty minutes post injection, PET imaging was performed acquiring approximately 50 million counts per mouse. As with the ^18^F-SynVesT-1 PET data, ^18^F-FDG PET images were reconstructed using 3-dimensional ordered-subset expectation maximization (OSEM, 2 iterations, 16 subsets) followed by a maximum a posteriori probability (MAP) algorithm. CT attenuation, scatter and decay correction were applied to all datasets. The PET and CT images were also reconstructed at the spatial resolutions of 128 × 128 × 159 (0.78 × 0.78 × 0.8 mm) and 480 × 480 × 635 (0.21 × 0.21 × 0.21 mm), respectively.

The Waxholm MR atlas (23) was used to identify the hippocampus, neocortex, amygdala, ventral thalamus, lateral thalamus, globus pallidus, caudate putamen, hypothalamus, and accumbens. After upsampling the PET images to match the resolution of the CT images, brain masks were semi-automatically generated by thresholding the CT image in MATLAB to include brain tissue and were refined using subsequent image dilation and filing steps as needed. All brain masks were visually confirmed for each subject. The Waxholm MR atlas was individually registered to the CT brain masks of each subject using affine registration in MATLAB (*imregtform*). Then, the registered Waxholm atlases were used to compute SUV values for each brain region.

### MR Imaging Acquisition, Data Preprocessing, and Analysis

Following PET imaging, animals were transcardially perfused with 4% paraformaldehyde and brains were excised for *ex vivo* MRI acquisition with a 4.7T Agilent MRI system and a 3.5 cm diameter quadrature volume RF coil. *Ex vivo* imaging and analysis, including standard data preprocessing, and study template generation, was performed as previously described (24). Multi-slice, diffusion-weighted spin echo images were used to acquire 10 non-diffusion-weighted images (b = 0 s/mm^2^) and 75 diffusion-weighted images (25 images: b=800 s/mm^2^; 50 images: b = 2,000 s/mm^2^) using non-collinear, diffusion-weighting directions. Diffusion imaging was performed with an echo time of 24.17/2,000 ms, field of view = 30 × 30 mm^2^, and matrix = 192 × 192 reconstructed to 256 × 256 for an isotropic voxel size of 0.25 mm over 2 signal averages. Multi-shell diffusion data were fit with the Microstructure Diffusion Toolbox (25) to the NODDI *ex vivo* model. An additional compartment of isotropic restriction was included to account for potential fixative effects as recommended (26). Tract-based spatial statistics (TBSS) was performed as previously described (21) with an FA threshold of 0.2 applied for skeleton generation, a permutation test with *n* = 252, multiple comparisons correction and threshold-free cluster enhancement; *p* < 0.05 was utilized as a threshold for significance. Regions of interest (ROIs), including the left and right hippocampus, frontal association cortex, and amygdala, were selected *a priori* and defined with a DTI-based mouse brain atlas (27).

### Statistical Analysis

Imaging sample size estimations are based on a Markov Chain Monte Carlo approach (17) and informed by our previously published work (18–21) with power analyses indicating observed effect size values of d = 2.5 or greater given low standard deviation between within-group replicates (σ between 0.01 and 0.001), thus validating sample sizes of 4 or greater replicates per group [significance level of 5% and power of 90% (11)]. Statistical differences between groups were performed using a two-tailed unpaired Student's *t*-test; *p* < 0.05 was established as the significance level. For MRI ROI analyses, a Student's *t-*test was used to determine the significance (*p* < 0.05) of ROI values between GF and gnotobiotic mice; statistically significant differences were determined after controlling for multiple comparisons with the Benjamini–Hochberg procedure (false discovery rate = 0.05).

## Results

### The Presence of a Functional Gut Microbiome Is Associated With Increased Neurite Density and Orientation Dispersion Compared to Germ-Free Mice

**Figure 1:**
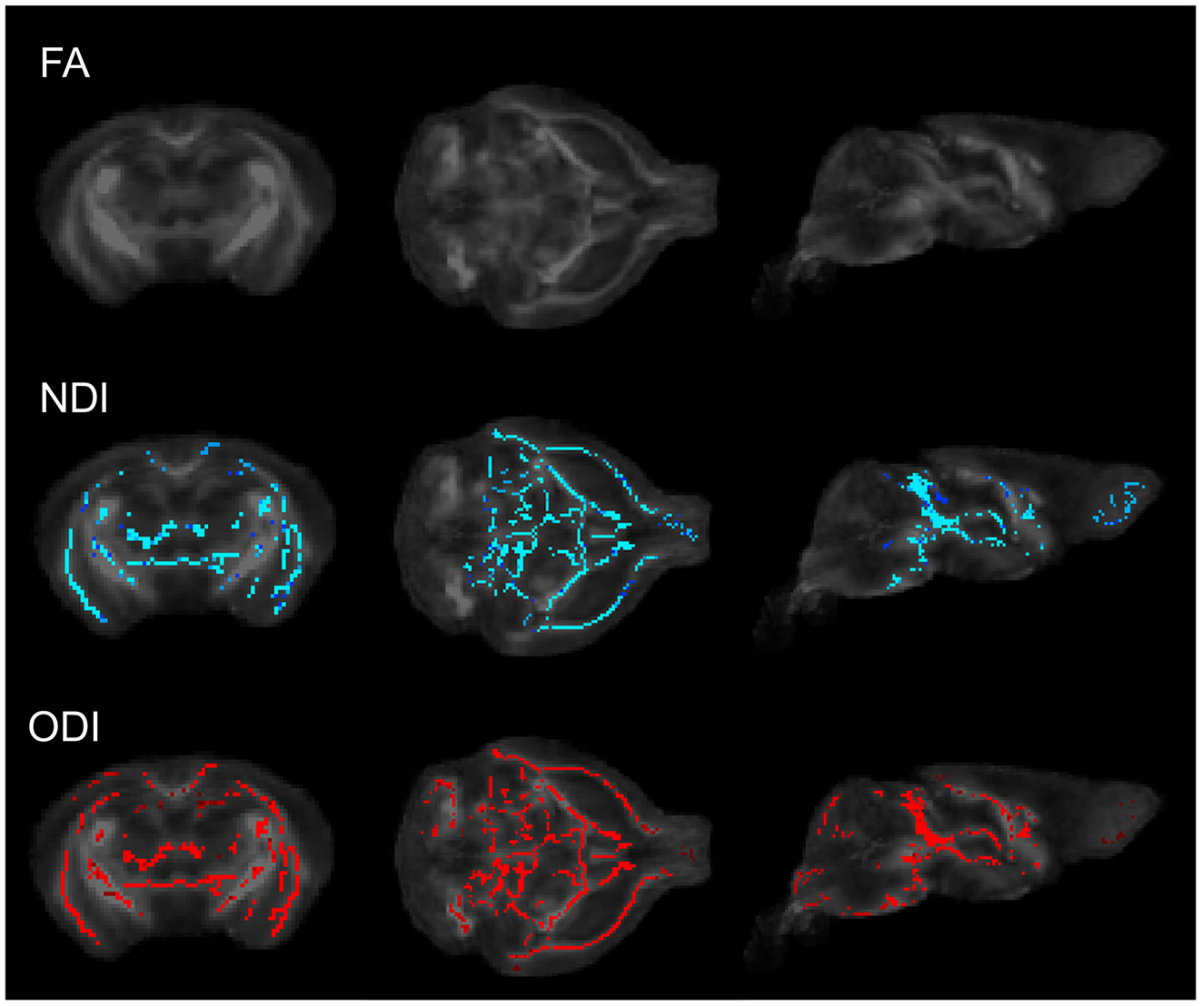
Coronal (left), axial (middle), and sagittal (right) TBSS plots of voxelwise changes in FA, NDI, and ODI between GF and HG P55 male animals. No significant voxelwise differences in fractional anisotropy (FA) were identified; however, TBSS analyses uncovered significant voxelwise reductions in both neurite density (NDI) and orientation dispersion (ODI) in GF mice when compared to HG mice. At P90, no significant voxelwise differences in NDI or ODI were detected between HG and GF animals.

To determine whether commonly employed diffusion weighted imaging techniques are sensitive to alterations in brain microstructure due to the presence of a gut microbiome, we first performed DTI and NODDI on male GF and HG mice. Whole-brain voxelwise tract-based spatial statistics (TBSS) analysis comparing the HG mice to age and sex-matched GF mice was performed at P55 and P90. No significant differences in fractional anisotropy (FA) were identified; however, TBSS studies uncovered significant voxelwise changes in mean diffusivity (MD) and a reduction in neurite density (NDI) and orientation dispersion (ODI) in GF mice when compared to HG mice at the P55 time point (Figure 1). By P90, TBSS showed similar global increases in MD, but no significant difference in NDI or ODI between humanized gnotobiotic (HG) and GF animals.

We next performed a region of interest (ROI) analysis to test the contribution of the gut microbiome to brain microstructure in ROIs salient to neuropsychiatric and neurocognitive illness. Three regions were selected *a priori* for further analysis: the hippocampus, frontal association cortex, and amygdala. Mean values of DTI and neurite indices were computed within each ROI (left and right) for each individual subject for a total of 6 calculated ROIs per subject. Our results match the TBSS findings with statistically significant increases in MD, reduced NDI, and reduced ODI found in most regions of GF mice when compared to HG animals (unpaired *t*-test; *p* < 0.05) (Table 1). Differences were again more evident in animals at P55 than at P90.

**Table 1:** Humanized gut microbiota contribute to significant changes in quantitative neural microstructure in salient regions implicated in neurologic and psychiatric illness.

**Age**	**DWI Measure**	**Hemi**.	**ROI**	**Mean** ±**SEM**		***p*-value**
				**Germ-free**	**Gnotobiotic**	
P55	FA	Left	HC	0.18315 ± 0.00530	0.18546 ± 0.00235	0.363
			FAC	0.15794 ± 0.00305	0.15979 ± 0.00271	0.345
			Amg	0.18017 ± 0.00344	0.17920 ± 0.00725	0.458
		Right	HC	0.18743 ± 0.00328	0.18871 ± 0.00258	0.393
			FAC	0.16682 ± 0.00501	0.15997 ± 0.00215	0.142
			Amg	0.19596 ± 0.00331	0.19550 ± 0.00253	0.461
	MD	Left	HC	0.38320 ± 0.00389	0.34560 ± 0.00545	<0.01*
			FAC	0.36760 ± 0.00570	0.32140 ± 0.00906	<0.01*
			Amg	0.36620 ± 0.00532	0.32980 ± 0.01027	<0.05*
		Right	HC	0.39140 ± 0.00430	0.34760 ± 0.00569	<0.01*
			FAC	0.36600 ± 0.00789	0.33640 ± 0.00658	<0.05*
			Amg	0.37100 ± 0.01221	0.32700 ± 0.00509	<0.01*
	NDI	Left	HC	0.27929 ± 0.00401	0.33678 ± 0.00871	<0.01*
			FAC	0.33005 ± 0.01774	0.40784 ± 0.02171	<0.05*
			Amg	0.31323 ± 0.00812	0.36850 ± 0.01866	<0.05*
		Right	HC	0.26832 ± 0.00641	0.33041 ± 0.00768	<0.01*
			FAC	0.33399 ± 0.03864	0.39707 ± 0.02270	0.117
			Amg	0.31268 ± 0.00634	0.36305 ± 0.00693	<0.01*
	ODI	Left	HC	0.28833 ± 0.01104	0.33706 ± 0.00446	<0.01*
			FAC	0.35944 ± 0.00625	0.43192 ± 0.01841	<0.01*
			Amg	0.34415 ± 0.01085	0.38631 ± 0.02160	0.075
		Right	HC	0.26686 ± 0.00623	0.32940 ± 0.00633	<0.01*
			FAC	0.32640 ± 0.01327	0.41286 ± 0.01424	<0.01*
			Amg	0.30175 ± 0.01577	0.36514 ± 0.00894	<0.01*
P90	FA	Left	HC	0.17947 ± 0.00321	0.17692 ± 0.00664	0.277
			FAC	0.14894 ± 0.00259	0.15253 ± 0.00441	0.172
			Amg	0.16868 ± 0.00484	0.16050 ± 0.00688	0.357
		Right	HC	0.18248 ± 0.00461	0.17599 ± 0.00305	0.178
			FAC	0.15173 ± 0.00193	0.14860 ± 0.00127	0.373
			Amg	0.18298 ± 0.00834	0.17442 ± 0.00324	0.218
	MD	Left	HC	0.45550 ± 0.00669	0.42325 ± 0.00239	<0.01*
			FAC	0.40650 ± 0.00462	0.37500 ± 0.00628	<0.01*
			Amg	0.48417 ± 0.01220	0.47075 ± 0.02176	0.175
		Right	HC	0.46800 ± 0.00870	0.43800 ± 0.01968	0.117
			FAC	0.39873 ± 0.01026	0.36650 ± 0.01538	*** <0.05***
			Amg	0.49067 ± 0.01766	0.46625 ± 0.00872	0.085
	NDI	Left	HC	0.37533 ± 0.00123	0.38404 ± 0.00123	<0.01*
			FAC	0.39376 ± 0.00464	0.40728 ± 0.00653	0.067
			Amg	0.37361 ± 0.00338	0.38199 ± 0.00777	0.144
		Right	HC	0.37332 ± 0.00051	0.38896 ± 0.00543	<0.05*
			FAC	0.39929 ± 0.01250	0.44848 ± 0.02088	<0.05*
			Amg	0.37223 ± 0.00378	0.38086 ± 0.00313	*** <0.05***
	ODI	Left	HC	0.24198 ± 0.00439	0.26345 ± 0.00832	*** <0.05***
			FAC	0.30100 ± 0.00383	0.35007 ± 0.00969	<0.01*
			Amg	0.25615 ± 0.00555	0.27611 ± 0.01576	0.196
		Right	HC	0.23252 ± 0.00841	0.25692 ± 0.00831	0.089
			FAC	0.30288 ± 0.01264	0.36021 ± 0.01150	<0.05*
			Amg	0.22202 ± 0.00796	0.25563 ± 0.00597	*** <0.05***

*All values are mean ± standard error of the mean (SEM). Units of measure for FA, MD, NDI, and ODI are [10^−3^ mm^2^/s]. Bolded and italicized p-values are statistically significant. Starred p-values are statistically significant after controlling the false discovery rate with the Benjamini-Hochberg procedure (false discovery rate = 0.05). Regions of interest (ROIs) correspond to ROIs defined from the Center for in vivo Microscopy (CIVM) mouse brain atlas. Ages of P55 and P90 animals are 45 and 90 days post-natal, respectively. P55 germ-free (n = 5) and gnotobiotic (n = 5) and P90 germ-free (n = 4) and gnotobiotic (n = 4) animals are all male. Hemi, hemisphere; FA, fractional anisotropy; NDI, neurite density index; ODI, orientation dispersion index; HC, hippocampus; FAC, frontal associations cortex; Amg, amygdala*.

### The Presence of a Functional Gut Microbiome Is Associated With Decreased Synaptic Density Compared to Germ-Free Mice

**Figure 2:**
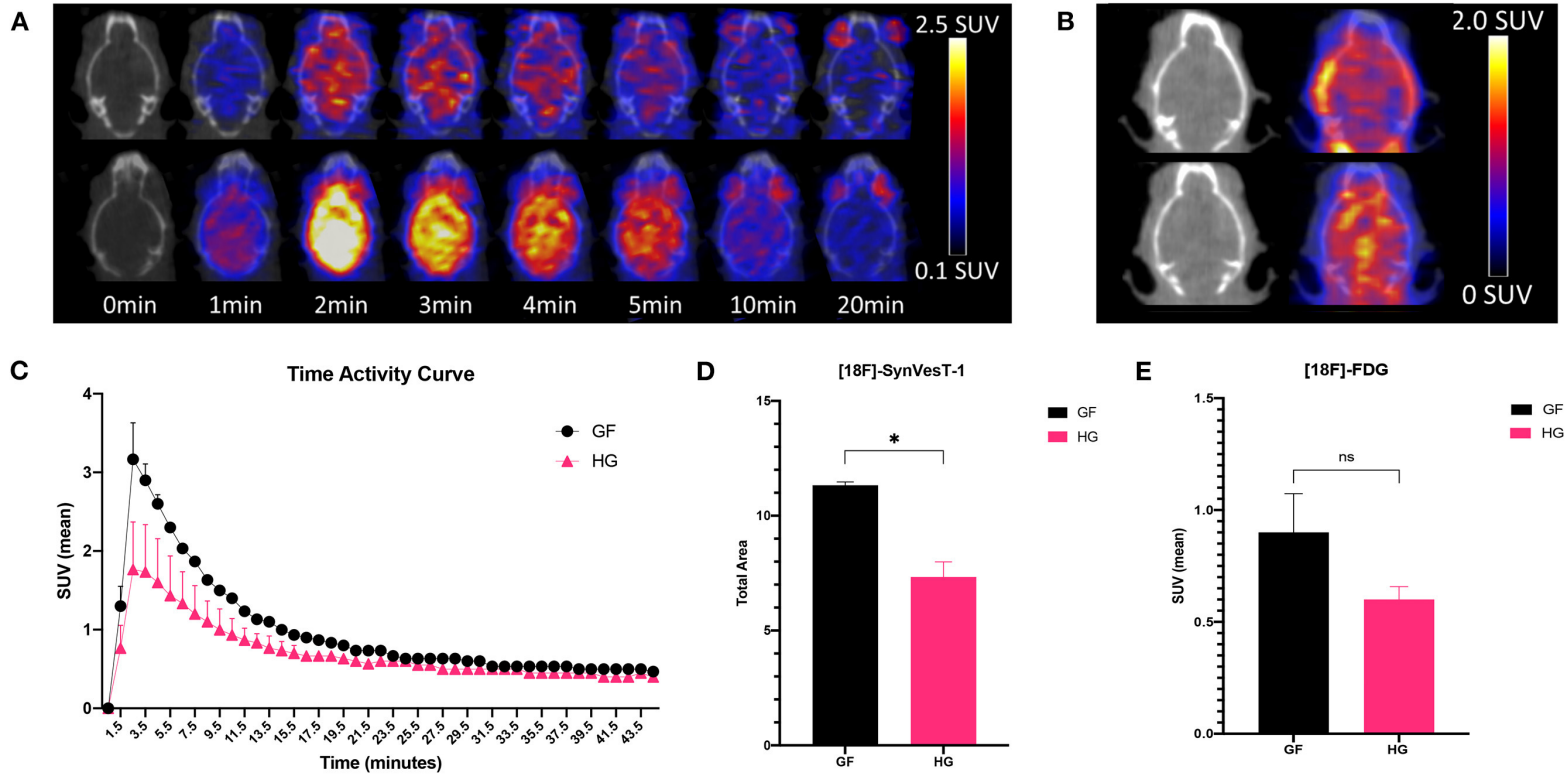
**(A)** Fused axial PET/CT images of male P90 HG (top row) and GF (bottom row) mice following the injection of ^18^F-SynVesT-1 demonstrating the qualitative higher uptake of the SynVesT-1 radiotracer in GF mice. **(B)** Static ^18^F-FDG images of HG (top row) and GF (bottom row). **(C)** Time-activity curves (mean ± SD) of male GF and HG P90 mice. The area under the curve was calculated for the peak stabilized interval (4.5–10.5 min) for ^18^F-SynVesT-1 **(D)** and ^18^F-FDG **(E)**. HG, humanized gnotobiotic mice; GF, germ-free; *p< *0.0*5.

The effect of gut microbiota on synaptic density was next analyzed via *in vivo* PET/CT imaging with ^18^F-SynVesT-1 at P90 (Figures 2A,B). ^18^F-FDG PET imaging was also performed to examine potential metabolic differences between GF and HG animals. Static ^18^F-FDG imaging was obtained 60 min after injection of the radiotracer; dynamic ^18^F-SynVesT-1 imaging was obtained over 45 min. The time-activity curve of ^18^F-SynVesT-1 shows a rapid increase, peaking at ~ 2 min, followed by clearance of the radiotracer by 20 min (Figure 2C). Area under the curve (AUC) analysis indicates significantly reduced SUV_(mean)_ in HG mice compared to the GF mice (Figure 2D). There was no significant difference in the uptake of ^18^F-FDG (Figure 2E). In parallel to our diffusion-weighted imaging ROI analysis, the hippocampus, neocortex, and amygdala were selected *a priori* for ^18^F-SynVesT-1 binding ROI analysis, while the ventral thalamus, lateral thalamus, globus pallidus, caudate putamen, hypothalamus and accumbens were further selected for exploratory analysis. HG mice demonstrated reduced SV2A binding with significantly reduced SUV_(mean)_ in all regions except for the caudate putamen (unpaired *t*-test; *p* < 0.05) (Table 2).

**Table 2:** Gut microbiota are associated with significant alterations in synaptic density.

**^**18**^F-SynVesT-1**	**ROI**	**Mean (±SEM)**		***p-*value**
		**Germ-free**	**Humanized gnotobiotic**	
SUV (mean)	HC	0.97565 (±0.04420)	0.76368 (±0.04091)	*** <0.05***
	NC	0.82295 (±0.03747)	0.67343 (±0.03653)	*** <0.05***
	AM	0.90888 (±0.02732)	0.73379 (±0.04848)	*** <0.05***
	VT	1.04453 (±0.06381)	0.79085 (±0.03936)	*** <0.05***
	LT	1.08326 (±0.06756)	0.80882 (±0.04963)	*** <0.05***
	GP	1.03449 (±0.06282)	0.80712 (±0.04187)	*** <0.05***
	CP	1.01670 (±0.07021)	0.79078 (±0.04224)	0.051
	HT	0.98326 (±0.05794)	0.76001 (±0.03679)	*** <0.05***
	AC	1.08539 (±0.07685)	0.81797 (±0.04625)	*** <0.05***
SUVR (cerebellum)	HC	1.44382 (±0.06410)	1.24986 (±0.02023)	*** <0.05***
	NC	1.21725 (±0.04783)	1.10206 (±0.01726)	0.086
	AM	1.34478 (±0.03501)	1.19967 (±0.03569)	*** <0.05***
	VT	1.54504 (±0.08755)	1.29514 (±0.02959)	0.054
	LT	1.60232 (±0.09286)	1.32233 (±0.01354)	*** <0.05***
	GP	1.53034 (±0.08726)	1.32165 (±0.03408)	0.090
	CP	1.50353 (±0.09553)	1.29435 (±0.02525)	0.102
	HT	1.45480 (±0.08210)	1.24527 (±0.03796)	0.081
	AC	1.60566 (±0.10849)	1.33834 (±0.02545)	0.074

*All values are mean ± standard error of the mean. Unit of measure is g/mL. Bolded and italicized p values are statistically significant. HC, hippocampus; NC, neocortex; AM, amygdala; VT, ventral thalamus; LT, lateral thalamus; GP, globus pallidus; CP, caudate putamen; HT, hypothalamus; AC, accumbens. HC, NC, and A were examined a priori, whereas the other ROIs were chosen for exploratory analysis*.

## Discussion

Gut bacteria significantly influence numerous molecular and cellular processes (7–9, 28–35) and are also associated with a broad spectrum of neuropsychiatric illnesses (36–40). With growing recognition of the salience of the gut microbiota in neurologic and neuropsychiatric health, there is not only an intense interest to more fully understand the gut microbiota-brain axis and its relationship to psychiatric illness but also to uncover the biological mechanisms that lead to the psychiatric disease state. However, significant barriers currently limit our ability to translate emerging scientific insights about gut microbiota effects on psychiatric health into clinical populations. These include a limited understanding of the contribution of gut microbiota to baseline measures of neural microstructure or brain function and the absence of a clinically accessible method to quantitatively evaluate the changes that occur in the brain due to alterations in the gut microbiota. To address these shortcomings, we have demonstrated the potential utility of ^18^F-SynVesT-1/NODDI PET/MRI as a novel multimodal neuroimaging method that can be utilized to acquire quantitative measures of structural (neurite density and morphology) and synaptic density changes attributable to the presence of an intact functional gut microbiome. We further show that GF mice harbor reduced neurite density and reduced orientation dispersion when compared to mice with a humanized gnotobiotic gut microbiome and additionally demonstrate GF mice possess increased synaptic density that is potentially attributable to poor synaptic pruning in the absence of a functional gut microbiome (9).

Although there are no directly comparable studies in the literature, our results confirm findings from previous animal studies that indicate that the gut microbiome is crucial for normal morphological development of neurons and synaptic pruning. Luczynski et al. (12) showed that the neurons of the hippocampus in germ-free mice had shorter neurites with less branching and thinner spines at a P63-70 time point, which is compatible with our findings of reduced neurite density and orientation dispersion in germ-free mice. Our PET findings are also compatible with recent work finding that germ-free mice over-express synapse-related genes, which leads to an abnormal increase in synapse formation (41). As previous neuroanatomical and cellular studies of the developing central nervous system are consistent with our neuroimaging findings, multiparametric PET/MR studies with ^18^F-SynVesT-1/NODDI are thus well-positioned to explore gut microbial influences on brain microstructure and synaptic density.

Our findings also raise important considerations for the neuroimaging of psychiatric illness and rigor and reproducibility of such research. A second but no less important conclusion of our results is the finding that gut colonization significantly influences quantitative neuroimaging measures of brain structure and function. Diffusion tensor imaging and emerging multicompartment diffusion imaging methods such as NODDI are widely used to study neural microstructure in neurologic and psychiatric illness; likewise, new SV2A synaptic PET radiotracers such as ^11^C-UCB-J and ^18^F-SynVesT-1 are rapidly emerging as important imaging tools to examine important biological correlates underlying the pathogenesis of schizophrenia and other psychiatric illnesses (42). Although we present and demonstrate ^18^F-SynVesT-1/NODDI PET/MRI as a multiparametric technique to capture salient structural and functional changes attributable to the presence of a functional gut microbiome, conversely and critically, it is also conceivable that psychiatric neuroimaging studies and imaging endophenotypes of psychiatric illness may be unknowingly affected by metagenomic effects that can further challenge efforts to identify and refine candidate neuroimaging biomarkers across a wide variety of diseases, especially those with a known connection to gut microbial dysbiosis.

Our study has limitations. One limitation is our abridged PET analysis. Ideally, binding potential of the SynVesT-1 tracer would be quantified in regions of interest through tracer kinetic modeling with metabolite correction following arterial blood sampling, which would be beyond the scope of a pilot study. In its place, we compared integrated time-activity curves at early time points due to fast tracer kinetics and expected high metabolite fractions at later time points as has been reported in other similar SV2A tracers (43). Likewise, reference region analysis using the centrum semiovale (white matter, as previously reported in human studies) is precluded in this murine study due to limited scanner resolution. Kinetic modeling with arterial blood sampling will be pursued in follow-up studies. An additional limitation is that our results do not provide insight into how future studies might discriminate the degree and extent observed structural and synaptic changes might be attributable to either the underlying disease process or unaccounted metagenomic contributions. Careful study design incorporating metagenomic data as potential study confounds will be needed to control and account for these important contributors to measured brain microstructure.

## Conclusion

In conclusion, we present initial pilot data for ^18^F-SynVesT-1/NODDI PET/MRI as a clinically accessible multiparametric neuroimaging approach to acquire both structural and synaptic brain measures that are quantitative, complementary, and reflective of brain changes attributable to the presence of a gut microbiome. Our work, for the first time, importantly highlights the sensitivity of commonly employed neuroimaging techniques (e.g., diffusion MRI) to non-genetic and environmental factors like the gut microbiome, which can potentially have a crucial and outsized effect in neuroimaging studies of the brain. This raises the potential need to incorporate such factors into future study designs if we are to achieve precision imaging in diseases of the CNS and to build greater rigor and reproducibility into quantitative neuroimaging research. With growing evidence of the role of the gut microbiome in early neurodevelopment and neuropsychiatric illness, ^18^F-SynVesT-1/NODDI PET/MRI can be employed to not only to further examine the biological underpinnings of the microbiome-gut-brain axis, but also to improve clinical diagnostic accuracy, patient risk stratification, and therapeutic monitoring for neuropsychiatric disorders.

## Data Availability Statement

The raw data supporting the conclusions of this article will be made available by the authors, without undue reservation.

## Ethics Statement

The animal study was reviewed and approved by University of Wisconsin Institutional Animal Care and Use Committee (IACUC).

## Author Contributions

SY, AP, and J-PY wrote the manuscript. SY, AP, JJ, BB, NS, and EV performed all experimental work. SY, AP, JW, TB, AM, FR, and J-PY interpreted the experimental data. J-PY is the guarantor of this work, and as such, had full access to all the data in the study and assumes responsibility for the integrity of the data and the accuracy of the data analyses. All authors contributed to the article and approved the submitted version.

## Funding

This work was supported by the Department of Radiology at the University of Wisconsin School of Medicine and Public Health and by the Brain and Behavior Research Foundation (NARSAD) Young Investigator Grant. Additional support was provided by the Clinical and Translational Science Award (CTSA) program, through the NIH National Center for Advancing Translational Sciences (NCATS) (UL1TR002373). SY was supported by the NIH/NIA award number F30AG066329. The content is solely the responsibility of the authors and does not necessarily represent the official views of the NIH.

## Conflict of Interest

The authors declare that the research was conducted in the absence of any commercial or financial relationships that could be construed as a potential conflict of interest.

## Publisher's Note

All claims expressed in this article are solely those of the authors and do not necessarily represent those of their affiliated organizations, or those of the publisher, the editors and the reviewers. Any product that may be evaluated in this article, or claim that may be made by its manufacturer, is not guaranteed or endorsed by the publisher.
